# How long does it take patients to find a new primary care physician when theirs retires: a population-based, longitudinal study

**DOI:** 10.1186/s12960-021-00633-9

**Published:** 2021-07-23

**Authors:** Lindsay Hedden, Megan A. Ahuja, M. Ruth Lavergne, Kimberlyn M. McGrail, Michael R. Law, Lucy Cheng, Morris L. Barer

**Affiliations:** 1 Faculty of Health Sciences, Simon Fraser University, Blusson Hall, Room 11300, 8888 University Drive, Burnaby, BC V5A 1S6 Canada; 2 Centre for Health Services and Policy Research, School of Population and Public Health, University of British Columbia, 201-2206 East Mall, Vancouver, BC V6T 1Z3 Canada

**Keywords:** Physician retirement, Primary care, Workforce planning, Majority source of care, Access

## Abstract

**Background:**

The retirement of a family physician can represent a challenge in accessibility and continuity of care for patients. In this population-based, longitudinal cohort study, we assess whether and how long it takes for patients to find a new majority source of primary care (MSOC) when theirs retires, and we investigate the effect of demographic and clinical characteristics on this process.

**Methods:**

We used provincial health insurance records to identify the complete cohort of patients whose majority source of care left clinical practice in either 2007/2008 or 2008/2009 and then calculated the number of days between their last visit with their original MSOC and their first visit with their new one. We compared the clinical and sociodemographic characteristics of patients who did and did not find a new MSOC in the three years following their original physician’s retirement using Chi-square and Fisher’s exact test. We also used Cox proportional hazards models to determine the adjusted association between patient age, sex, socioeconomic status, location and morbidity level (measured using Johns Hopkins’ Aggregated Diagnostic Groupings), and time to finding a new primary care physician. We produce survival curves stratified by patient age, sex, income and morbidity.

**Results:**

Fifty-four percent of patients found a new MSOC within the first 12 months following their physician’s retirement. Six percent of patients still had not found a new physician after 36 months. Patients who were older and had higher levels of morbidity were more likely to find a new MSOC and found one faster than younger, healthier patients. Patients located in more urban regional health authorities also took longer to find a new MSOC compared to those in rural areas.

**Conclusions:**

Primary care physician retirements represent a potential threat to accessibility; patients followed in this study took more than a year on average to find a new MSOC after their physician retired. Providing programmatic support to retiring physicians and their patients, as well as addressing shortages of longitudinal primary care more broadly could help to ensure smoother retirement transitions.

## Background

There are longstanding concerns about the potential impact of primary care physician retirements on patient access to and continuity of care, and on service supply more broadly [1–8]. While considerable research has been done on the services that older physicians provide as they age and transition out of the workforce [1, 2, 9–13], very little attention has been given to how physician retirement affects patients [7]. Existing work suggests that disruption to longitudinal relationships between patients and physicians has been associated with difficulty accessing care [14], emotional distress [14, 15], poorer health outcomes [8], and decreased use of downstream primary care services [7, 8].

In 2019, eighteen percent of British Columbians and 15% of Canadians overall stated that they did not have a regular source of primary care [16]. Existing research suggests that patients without a regular source of primary care are more likely to rely on walk-in clinics or emergency departments [17–19]. This has a negative effect on care continuity and coordination [19–23], and access to preventive services [24–26].

Physician retirements add an additional challenge, as they mean that some patients who have a regular source of care then lose it. In a recent survey of patients who were actively seeking a primary care physician, 20% of respondents reported that they were looking because their physician had either retired or moved [27]. While finding a family physician may not be a high priority for young, healthy patients, it is critical for many, particularly those who have chronic conditions that require regular monitoring [27]. Like the broader population of unattached patients, patients of retired physicians reported using walk-in clinics or emergency departments more frequently [28]. They also reported concerns about poor continuity and poor access to preventative care [28].

Many provincial medical associations and state medical boards have published guidelines and recommendations for physicians who are considering retiring from clinical practice (e.g., [15, 33–36]) These guidelines include notifying patients of their leave date, whether another physician is taking over their practice, or providing a list of physicians taking new patients in the clinic or community [15, 35, 36]. Physicians are concerned about the negative impact their retirement may have on their patients. Several studies have reported that a feeling of responsibility for their patients (concerns about what will happen to the patients after the physician retires) keeps many physicians in practice longer than might otherwise have been the case [37–42].

The possible effect of physician retirement on access to primary care services across Canada in general, and in British Columbia (BC) in particular, has received some academic and media attention [1–8]. This paper expands on this existing literature by examining the use of primary care services by patients whose primary care physician retires. We describe both the periods preceding and following the retirement. Our research objectives are: (1) to measure the amount of time it takes for patients to find a new primary care physician; and (2) to assess whether time to finding a new primary care physician is associated with specific clinical and sociodemographic characteristics.

## Methods

### Approach

For this population-based, retrospective cohort study, we used administrative physician fee-for-service payment data to identify a cohort of patients and their primary care physicians, determine which physicians retired over the study period, and examine primary care interactions for their patients before and following that retirement. We then compared primary care interactions with a cohort of patients whose physicians did not retire.

### Data sources

Health care in Canada is primarily publicly funded (through a variety of tax revenues), and privately delivered. Most primary care physicians run clinics as independent, small businesses and bill provincial health insurance plans (e.g., British Columbia Medical Services Plan (MSP)) on a fee-for-service basis; however, alternative models of payment such as capitation are becoming increasingly common in some provinces (though not in BC) [43]. Primary care physicians in Canada serve a coordinating and gate-keeping function; patients require a referral from a primary care physician to see a specialist. BC is divided into five regional health authorities that are responsible for service delivery within their respective geographic regions.

We used the British Columbia (MSP) Consolidation file [44] to identify patients who were registered to receive MSP-insured services for the entire period of 2005/2006 through 2011/2012. The Consolidation file also contains basic patient demographic data including age, sex, regional health authority (region) of residence, and income quintile.

Using unique patient-level identifiers, we linked the Consolidation file data to the MSP Physician Payment Database [45], which is a record of all physician–patient interactions billed on a fee-for-service (FFS) basis. These interactions (visits) include any face-to-face consultation between a physician and patient regardless of location (e.g., urgent care or community clinic). We used this data source to identify the Majority Source of Care (MSOC) for each patient, defined as the physician who provided more than 50% of their primary care contacts. To calculate this measure, patients must have had a minimum of three primary care visits in a single calendar year. This was necessary to ensure more accurate assignment of physicians to patients; it also permitted us to focus on a population likely to be most affected by any changes in primary care interactions surrounding retirement. We identified patients’ MSOCs only for 2005/2006 and 2006/2007 to avoid artificially limiting our sample to very high users.

We supplemented MSP FFS billings with data from the Alternative Payment Plan database, which tracks payments to physicians outside of the traditional FFS model. This allowed us to confirm that physicians who ceased billing MSP did in fact retire, rather than move to an alternative payment model.

We also used data from the MSP Physician Payment Database [45] and from hospital separations data [46] to quantify the level of morbidity for each patient, using Johns Hopkins Aggregated Diagnostic Groupings (ADGs). ADGs are generated using the International Classification of Diseases, 9th and 10th Revisions, diagnostic codes attached to specific health services utilization [47, 48]. We focused on eight of 32 possible ADGs that are considered to be “major conditions” [47, 48].

Finally, we used data from the College of Physicians and Surgeons of BC physician registry [49] as a source for physician demographic data including age, sex, and training location (Canada or international) of each MSOC physician.

### Study cohort

We included all individuals in BC who were continuously registered to receive MSP-insured services between 2005/2006 and 2011/2012, and who could be identified as having the same MSOC physician for 2005/2006 and 2006/2007. Patients with fewer than three visits annually were not assigned an MSOC and were therefore excluded.

Eligible physicians (i.e. those who might reasonably be considered as possible retirement candidates) were defined as those who were over age 50 in 2005/2006, had eligible practice licenses (i.e. did not practice out-of-province, opt out of public practice, or have their license suspended), billed FFS during the study period, and were MSOC for at least one patient. We chose age 50 as the cut-off to ensure we were not mistakenly capturing physicians leaving practice for reasons other than retirement [50]. We defined retirement as a complete cessation of FFS and non-FFS (alternative payment plan) billings consistent with previously published work in this area [50, 51].

The data for both physicians and patients were stripped of identifiers, and then labeled with a unique study identifier in order to facilitate data set linkage and analysis without the risk of identification. The University of British Columbia’s Research Ethics Board granted ethics approval for this project and data linkage services were provided by Population Data BC. All inferences, opinions, and conclusions drawn in this manuscript are those of the authors, and do not reflect the opinions or policies of the Data Stewards.

### Outcomes

Our main outcomes of interest were (1) whether or not patients whose MSOC retired in 2007/2008 or 2008/2009 found a new MSOC before the end of the study period; and (2) how long that process took. For each patient whose MSOC retired, we computed time in days between the last visit with them, and the first visit with a new primary care physician who eventually became the patient’s new MSOC.

We conducted descriptive analyses to determine whether patients whose MSOC physician retired are statistically different from those whose physician did not retire (according to age, sex, income quintile and morbidity level) using Chi-square and Fisher’s exact tests. We used the same approach to compare individuals who found a new MSOC with those who did not.

### Exposures

We used multivariable Cox proportional hazards regression to determine the association between demographic factors, age, sex, socioeconomic status, location and aggregated clinical groups, and time to finding a new primary care physician after their original physician retired. We produce survival analysis graphs stratified by sex, age group, morbidity level, and income quintile, using the date of physician retirement as time zero. We used SAS statistical software to complete our analyses.

## Results

**Fig. 1:**
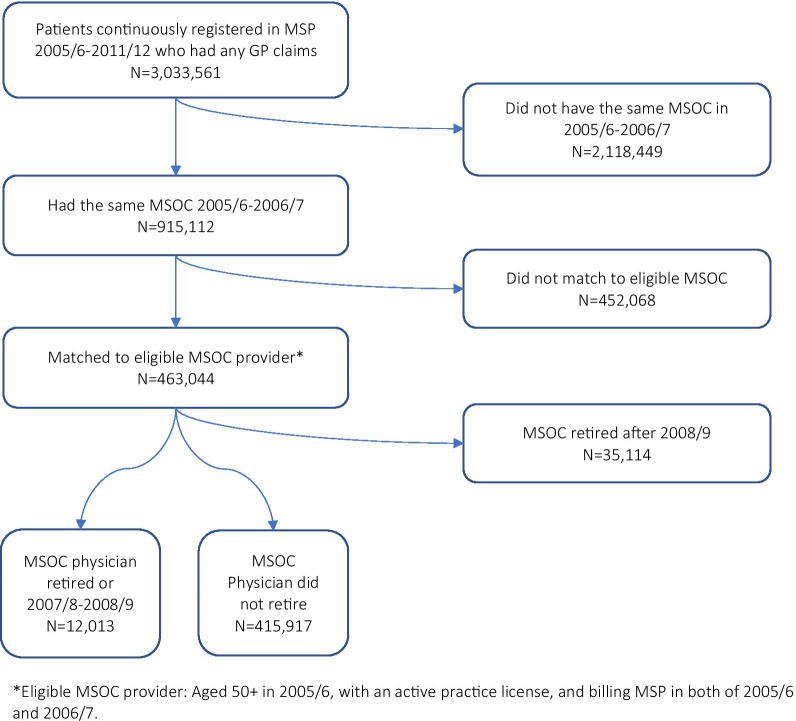
Patient cohort selection

We identified 463,044 patients who were continuously registered with MSP between 2005/2006 and 2011/2012 and who had the same MSOC for 2005/2006 and 2006/2007 (Fig. 1). Of these, 12,013 patients had an MSOC who was over age 50 at the beginning of the study period, and who ceased clinical practice in either 2007/2008 or 2008/2009 (*N* = 68 unique physicians) (Table 1).^1^ A majority of the patients of retiring physicians were over age 50 (69.3%). Just over half were women (54.5%). Patients were equally distributed across the five income quintiles. Half had no major ADGs (52.1%) while 4.8% had three or more.

**Table 1:** Patient cohort characteristics

Characteristic (in 2005/2006)	Number (row %) of individuals			*P* value
	Patients with MSOC who retired (*N* = 12,013)	Patients with MSOC who did not retire (*N* = 415,917)	Total	
*Age*				< 0.0001
< 20	527 (4.4)	32,606 (7.8)	33,133	
20–29	454 (3.8)	21,361 (5.1)	21,815	
30–39	832 (6.9)	37,098 (8.9)	37,930	
40–49	1884 (15.7)	70,052 (16.8)	71,936	
50–59	2915 (24.3)	92,378 (22.2)	95,293	
60–69	2569 (21.4)	76,481 (18.4)	79,050	
70–79	1925 (16.0)	60,583 (14.6)	62,508	
80 +	907 (7.6)	25,358 (6.1)	26,265	
*Sex*				< 0.0001
Female	6548 (54.5)	236,570 (56.9)	243,118	
Male	5460 (45.5)	179,186 (43.1)	184,646	
Unknown*	≤ 5	≤ 161	166	
*Socioeconomic status*				
1—lowest	2517 (21.0)	82,914 (19.9)	85,431	< 0.0001
2	2450 (20.4)	85,796 (20.6)	88,246	
3	2164 (18.0)	83,600 (20.1)	85,764	
4	2267 (18.9)	79,805 (19.2)	82,072	
5—highest	2445 (20.4)	79,726 (19.2)	82,171	
Unknown	170 (1.4)	4076 (1.0)	4246	
*Health authority of residence*				< 0.0001
Interior health	2286 (19.0)	55,283 (13.3)	57,569	
Fraser health	3056 (25.4)	151,886 (36.5)	154,942	
Vancouver coastal health	2958 (24.6)	118,014 (28.4)	120,972	
Island health	2948 (24.5)	73,747 (17.7)	76,695	
Northern health	755 (6.3)	16,560 (4.0)	17,315	
*Major ADGs*				0.0005
0	6253 (52.1)	224,315 (53.9)	230,568	
1	3766 (31.4)	127,358 (30.6)	131,124	
2	1418 (11.8)	45,866 (11)	47,284	
3	429 (3.6)	13,744 (3.3)	14,173	
4 +	147 (1.2)	4634 (1.1)	4781	

*Precise numbers and percentages not included due to small cell counts (to preserve anonymity)

**Fig. 2:**
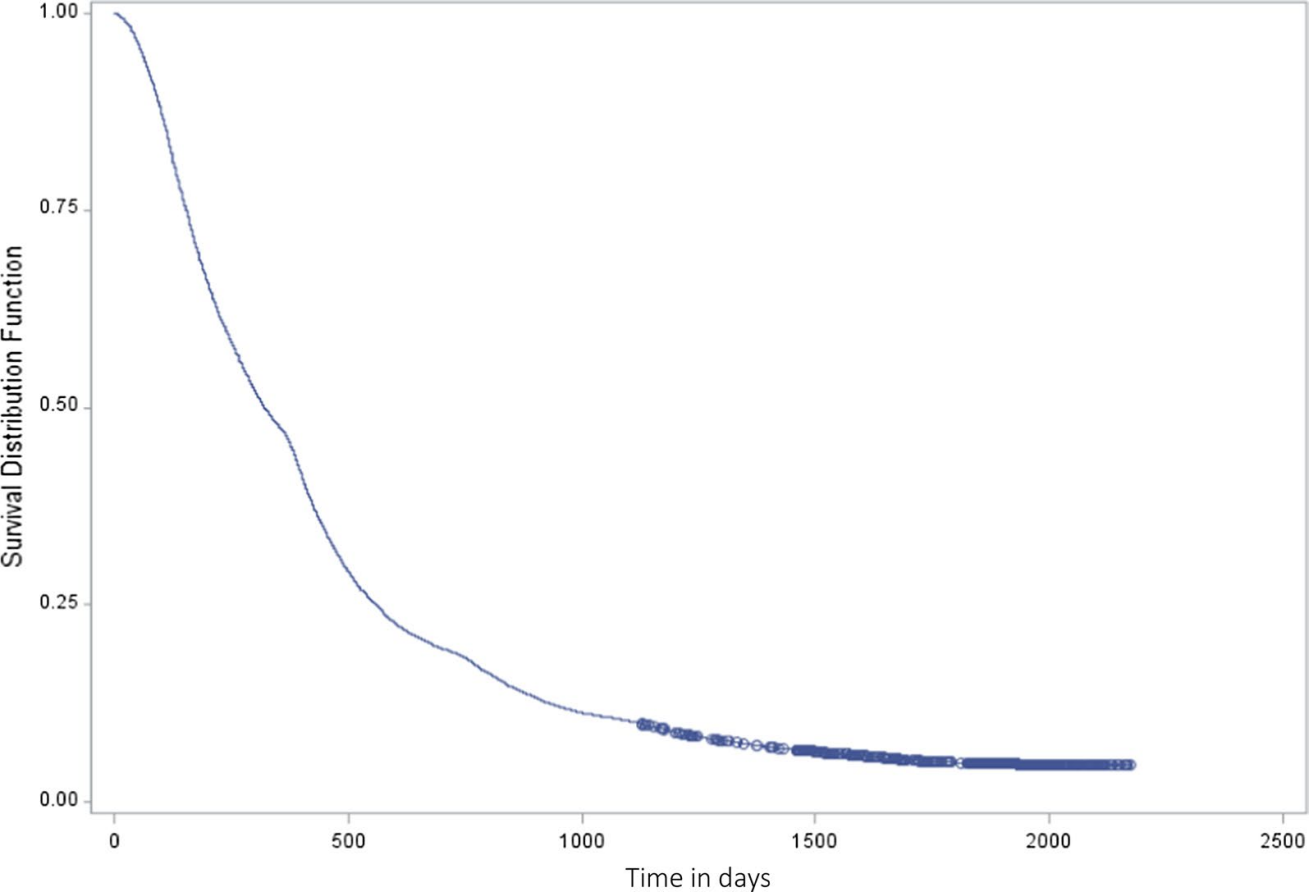
Time to finding a new MSOC among patients of retiring primary care practitioners

The vast majority (94.3%) of the patients found a new MSOC between when their original MSOC retired (in 2007/2008–2008/2009) and the end of the study period (2011/2012) (Table 2). However, 45.8% took longer than one year, and 5.7% still had not found one 36 months after their original MSOC retired (Fig. 2).

**Table 2:** Patients who found or did not find a new MSOC following their physician’s retirement

Characteristic (in 2005/2006)	Number (row %) of individuals			
	Patients who found new MSOC *N* = 11,377	Patients who did not find new MSOC *N* = 636	Total *N* = 12,013	*P* value
*Age*				
< 20	408 (3.6)	119 (18.7)	527	< 0.0001
20–29	387 (3.4)	67 (10.5)	454	
30–39	739 (6.5)	93 (14.6)	832	
40–49	1755 (15.4)	129 (20.3)	1884	
50–59	2800 (24.6)	115 (18.1)	2915	
60–69	2512 (22.1)	57 (9.0)	2569	
70–79	1896 (16.7)	29 (4.6)	1925	
80 +	880 (7.7)	27 (4.3)	907	
*Sex*				*p* = 0.0016
Female	6242 (54.9)	306 (48.1)	6548	
Male	5131 (45.1)	329 (51.7)	5460	
Unknown*	≤ 5	≤ 5	≤ 5	
*Socioeconomic status*				< 0.0001
1—lowest	2334 (20.5)	183 (28.8)	2517	
2	2327 (20.5)	123 (19.3)	2450	
3	2061 (18.1)	103 (16.2)	2164	
4	2157 (19.0)	110 (17.3)	2267	
5—highest	2341 (20.6)	104 (16.4)	2445	
Unknown	157 (1.4)	13 (2.0)	170	
*Major ADGs*				< 0.0001
0	5851 (51.4)	402 (63.2)	6253	
1	3603 (31.7)	163 (25.6)	3766	
2	1362 (12)	56 (8.8)	1418	
3	418 (3.7)	11 (1.7)	429	
4 + *	≥ 142	≤ 5	147	
*Health authority of residence*				*p* = 0.16
Interior health	2182 (19.2)	104 (16.4)	2286	
Fraser health	2882 (25.3)	174 (27.4)	3056	
Vancouver coastal health	2789 (24.5)	169 (26.6)	2958	
Island health	2805 (24.7)	143 (22.5)	2948	
Northern health	710 (94.04)	45 (5.96)	755	
*Time to finding new MSOC*				
< 6 months	3247 (28.5)			
6–12 months	2924 (25.7)			
12–18 months	2531 (22.3)			
18–24 months	970 (8.5)			
24–30 months	701 (6.2)			
30–36 months	357 (3.1)			
> 36 months	647 (5.7)			

*Precise numbers and percentages not included due to small cell counts (to preserve anonymity)

Compared to patients who successfully found a new MSOC, those who did not were more likely to be in the younger age categories (for example 18.7% versus 3.6% under age 20, *p* < 0.0001), male (51.7% versus 45.1%, *p* < 0.0001) and have fewer major ADGs (for example 63.2% versus 51.4 had no major ADGs, *p* < 0.0001). They were also more likely to be in the lowest income quintile (28.8% versus 20.5%, *p* < 0.0001).

**Fig. 3:**
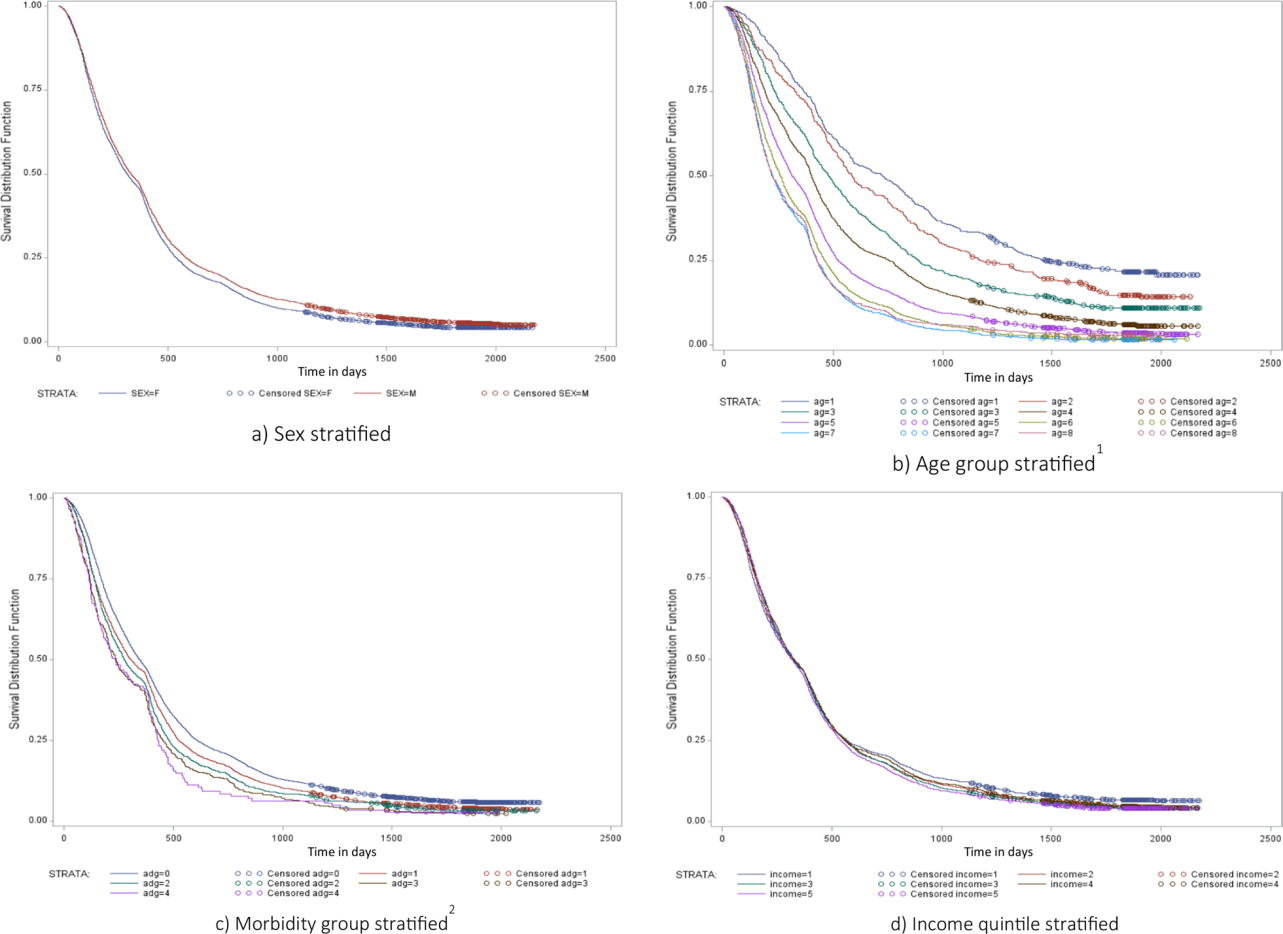
Time to finding to a new MSOC among patients of retiring primary care practitioners (stratified by sex, age, morbidity and income quantile)

In the time-dependent analysis and controlling for the effect of other factors, men had lower adjusted rates of finding a new MSOC compared to women (HR 0.92, 95% CI 0.88–0.95) (Table 3, Fig. 3). We observed a clear trend by age group, with individuals in the oldest age categories having the highest likelihood of finding a new MSOC compared to those in the lowest age category (e.g., individuals 70–79 HR 3.26, 95% CI 2.91–3.65). A patient’s regional health authority of residence was also associated with the odds of finding a new MSOC, with individuals located in two of the health authorities having less success with finding a new physician (Vancouver Coastal Health: HR 0.73, 95% CI 0.67–0.78; Vancouver Island: HR 0.86, 0.80–0.93); however regional primary care physician supply had no effect (HR 1.0, 95% CI 0.98–1.01).

**Table 3:** Adjusted hazard ratios from multivariable Cox regression

	Hazard ratio	95% CI	*P*-value
*Patient characteristics*			
Male sex	0.92	0.88–0.95	< 0.0001
Age group (reference = < 20)			
20–29	1.18	1.02–1.36	0.028
30–39	1.47	1.29–1.66	< 0.0001
40–49	1.85	1.65–2.06	< 0.0001
50–59	2.37	2.12–2.64	< 0.0001
60–69	2.92	2.61–3.26	< 0.0001
70–79	3.26	2.91–3.65	< 0.0001
> = 80	3.04	2.68–3.44	< 0.0001
*Health authority of residence (reference = interior health)*			
Fraser health	0.90	0.84–0.97	0.008
Vancouver coastal	0.73	0.67–0.78	< 0.0001
Vancouver Island	0.86	0.80–0.93	< 0.0001
Northern health	0.95	0.85–1.06	0.358
*Income quintile (reference = 1, lowest)*			
2	1.05	0.99–1.12	0.098
3	1.04	0.98–1.11	0.187
4	1.03	0.97–1.10	0.302
5 (highest)	1.07	1.00–1.13	0.036
*Major ADGs (reference = 0)*			
1	1.05	1.01–1.1	0.028
2	1.08	1.02–1.15	0.015
3	1.20	1.08–1.33	0.001
4 +	1.19	1.00–1.41	0.052
Regional primary care supply	1.00	0.98–1.01	0.470
*Physician characteristics*			
Male sex	0.90	0.85–0.96	0.001
Age group (reference = < 55)			
56–59	1.28	1.19–1.38	< 0.0001
60–64	1.36	1.28–1.45	< 0.0001
65–69	1.34	1.24–1.44	< 0.0001
70–74	1.25	1.15–1.35	< 0.0001
75 +	1.35	1.23–1.49	< 0.0001
Trained in Canada (vs international)	1.03	0.97–1.08	0.356

Rates of finding a new MSOC were positively associated with morbidity, with individuals having 3, or 4+ ADGs in particular having higher rates (3 Major ADGs: HR 1.20, 95% CI 1.08–1.33; 4+ Major ADGs: HR 1.19, 1.00–1.41). Compared with the lowest income quintile, patients in the highest income quintile had small but statistically significantly greater likelihood of finding a new MSOC (HR 1.07, 95%CI 1.00–1.13).

Physician characteristics also influenced time to finding a new MSOC. Patients whose original (retired) physician was male (HR 0.90, *p* = 0.001) had lower adjusted rates of finding a new MSOC, while patients whose retiring physicians were in any but the youngest age category had significantly higher rates (e.g., age 75 + HR 1.35, *p* < 0.0001). We observed no difference between physicians who trained internationally and those who trained in Canada.

## Discussion

To our knowledge, this is the first study that examines patients’ primary care interactions in the period following the retirement of their primary care physician, using a population-based approach. We found that while the vast majority of patients do go on to find a new MSOC, that process can take considerable time. Six percent of our sample had not found a family physician 36 months after the retirement of their original MSOC. Eighteen percent took between 18 and 36 months.

Older patients and those with more significant morbidity had higher rates of finding a new primary care physician, and found one more quickly, consistent with the suggestion that younger, healthier patients are less likely to make seeking out a new, consistent, source of primary care a priority, even within this cohort of patients with three or more visits per year [52]. Individuals in the lowest income quintile were somewhat less likely to find a new MSOC. While at first glance this may seem to be an artifact of fewer physicians providing longitudinal primary care in low-income areas, this relationship persisted in the Cox regression where regional primary care supply was accounted for. This finding raises questions about whether marginalized populations are less likely to seek out a new family physician, or less successful at doing so. There is evidence suggesting that the likelihood of having at least one primary care visit is independent of income [53, 54]; however, individuals who have low socioeconomic status are more likely to face discrimination when seeking out an appointment with a new primary care physician [55], suggesting that they still have relatively more challenges with accessing primary care may compared to individuals in higher income groups.

We found significant differences in the rates of individuals finding a new MSOC across regional health authorities, even when we accounted for differences in regional primary care capacity. Individuals in the largest (by population) and most urban health authority had the lowest time-adjusted rates of finding a new MSOC. On the surface, this finding seems contrary to the body of evidence suggesting that accessibility challenges are more salient in rural areas compared to urban ones; however, it may relate to greater access to walk-in clinics in urban centers in particular, and to the role these clinics play in shaping access to longitudinal primary care.

Several organizations have tools and programs designed to assist physicians with the process of retirement, one element of which is ensuring a smooth transition for their patients (for example [56, 57]). For example the B.C. General Practice Services Committee, through their broader *GP for Me* Initiative, offers a program that attempts to match new-to-practice physicians with retiring general practitioners, while also providing business coaching and support [57]. To our knowledge, however, neither this program nor any of the other supports offered, have been evaluated in terms of their ability to assist physicians with retirement, or ensure an easy transition for their patients.

Furthermore, given the more general ‘taut’ primary care supply situation in the province, retiring physicians’ ability to find younger primary care practitioners who are able to take on large numbers of new patients is likely to be an incredible challenge. Connection to a centralized waiting list is one potential solution that is being trialed in a number of Canadian provinces [58]. While there are wide differences in implementation of these lists, a consistent challenge among them is extensive waiting periods due to demand for attachment that far outstrips supply [59]. Lower rates of attachment of vulnerable patients, such as those with harmful substance use issues, mental health concerns, or chronic diseases are also a known concern with some implementations of this type of management strategy [59, 60].

### Limitations

While we believe that the use of administrative payment data is a strength of this study, allowing for a population-based, comprehensive analysis, the approach is not without its challenges. For example, our measure of retirement was necessarily blunt. The chosen data sources provide no ability to determine whether patients of retiring physicians had been looking for or transitioning to new MSOC before their original physician ceased clinical practice. Our measure of time to finding a new MSOC would certainly be an underestimate in such cases.

Our measure of attachment, majority source of care, is also relatively crude. It requires a minimum of three annual visits, which means that healthier patients who interact with the health system less often are under-represented. This is necessary to ensure more accurate assignment of physicians to patients and also focuses in on a population that may be most impacted by any changes in primary care interactions surrounding retirement. The MSOC measure does not, however, account for patients who are seen within a team-based model of practice, though these models were rare in BC in the time period represented.

Our patient cohort includes only individuals who were continuously registered with BC MSP for the full study period. Thus, patients who died or moved out of province were excluded. This may introduce an element of selection bias in that some of the sickest patients were likely to have been excluded.

The use of the Alternative Payments Database (APD) ensures that physicians who move from FFS to an alternative funding model are not misclassified as having retired. However, we have no ability to track individual patient interactions in the APD. This limitation artificially reduces the number of visits patients might have with a particular physician, in turn reducing the attachment (MSOC) rate. However, given that few physicians tend to transition to non-FFS contracts towards the end of their practice careers [61], we do not expect this would have affected our results. Furthermore, the APD was only available for analysis through 2011/2012, preventing us from assessing trends in more recent years.

As this research was grounded in administrative data, we cannot comment on patient perspectives or experiences with physician retirement or subsequent accessibility or continuity issues. This remains a critical knowledge gap and one that should be explored in future studies, with a particular focus on potential equity considerations for vulnerable populations.

## Conclusion

Primary care physician retirements represent a potential challenge to accessibility and continuity of care for patients, particularly in light of apparent systemic shortages in many provinces. We find that, on average, patients take more than a year to find a new regular source of primary care following the retirement of their original physician. Existing programs intended to smooth the retirement transition for physicians and for patients should be evaluated; however, we expect their efficacy is likely to be hampered by broader accessibly challenges. These can only be addressed through initiatives designed to increase capacity for longitudinal, community-based primary care.

## Data Availability

The data that support the findings of this study are available from Population Data BC but restrictions apply to the availability of these data, which were used under license for the current study, and so are not publicly available. Permission to access these data can be obtained from the data holders pending a completed data access request and ethics approval from the appropriate research ethics board. All inferences, opinions, and conclusions drawn in this manuscript are those of the authors, and do not reflect the opinions or policies of the Data Stewards.

## Abbreviations

ADGs: Aggregated diagnostic groupings
APD: Alternative payments database
BC: British Columbia
FFS: Fee-for-service
MSOC: Majority source of care
MSP: Medical services plan

## Footnotes

1 This implies an average of 173 MSOC patients per retiring physician. The fact that this number is small likely reflects the fact that the MSOC measure requires three visits per year, with the same physician providing at least two of those visits.
